# Effect of Thermal Aging on the Mechanical Properties of High Tenacity Polyester Yarn

**DOI:** 10.3390/ma14071666

**Published:** 2021-03-28

**Authors:** Tsegaye Sh. Lemmi, Marcin Barburski, Adam Kabziński, Krzysztof Frukacz

**Affiliations:** 1 Faculty of Material Technologies and Textile Design, Institute of Architecture of Textiles, Lodz University of Technology, 90-924 Lodz, Poland; Tsegaye.lemmi@dokt.p.lodz.pl; 2 Sempertrans Bełchatów Sp. z o.o., 97-427 Rogowiec, Poland; Adam.Kabzinski@semperitgroup.com (A.K.); Krzysztof.Frukacz@semperitgroup.com (K.F.)

**Keywords:** thermal aging, high tenacity, polyester fiber, mechanical properties, industrial yarn

## Abstract

Textile materials produced from a high tenacity industrial polyester fiber are most widely used in the mechanical rubber goods industry to reinforce conveyor belts, tire cords, and hoses. Reinforcement of textile rubber undergoes a vulcanization process to adhere the textile materials with the rubber and to enhance the physio-mechanical properties of the product. The vulcanization process has an influence on the textile material being used as a reinforcement. In this work, the effects of aging temperature and time on the high tenacity polyester yarn’s mechanical and surface structural properties were investigated. An experiment was carried out on a pre-activated high tenacity polyester yarn of different linear densities, by aging the yarn specimens under various aging temperatures of 140, 160, 200, and 220 °C for six, twelve, and thirty-five minutes of aging time. The tensile properties and surface structural change in the yarns pre- and post-aging were studied. The investigation illustrates that aging time and temperature influence the surface structure of the fiber, tenacity, and elongation properties of the yarn. Compared to unaged yarn, an almost five times higher percentage of elongation was obtained for the samples aged at 220 °C for 6 min, while the lowest tenacity was obtained for the sample subjected to aging under 220 °C for 35 min.

## 1. Introduction

Polyester fiber is the most widely used synthetic fiber in several areas of application [1]. As per a report published by the textile exchange in 2019, polyester fiber had 51.5 percent of the total global fiber production market share in 2018 [2]. The use of polyester fiber ranges from apparel and household textiles, to industrial and special textiles [3]. This work only focuses on industrial polyester yarns.

Based on the fiber’s morphological structure and properties, four primary industrial polyester yarns are commercially available. These yarns are high tenacity (HT) polyester yarn, high-modulus low-shrinkage (HMLS) polyester yarn, low-shrinkage (LS) polyester yarn, and super low-shrinkage (SLS) polyester yarn [4]. The availability of polyester (poly(ethylene terephthalate)) in a semi-crystalline or fully amorphous form attracts the attention of many researchers, and numerous studies have been conducted on the properties of poly(ethylene terephthalate) (PET). Samui et al. [4] studied the relationship between the morphology and the properties of industrial polyester yarns. The study shows that amorphous orientation is the key structural parameter that has an influence on the essential properties of the yarn. Additionally, in another article, Samui et al. [5] studied the relation between the dynamic and static properties of different types of industrial polyester yarns, with their structure and end applications. Martin et al. [6] investigated the effect of the physical aging of semi-crystalline poly(ethylene terephthalate) on the degree of crystallinity. Jing and Shanyuan [7] analyzed the modulus–strain curve of poly(ethylene terephthalate) and polyamide yarns in relation to physical structure change. Farhoodi et al. [8] studied the physical aging of semi-crystalline poly(ethylene terephthalate) at different aging temperatures using differential scanning calorimetry (DSC). The study signifies that the crystallization process of polymer chains can be enhanced as the aging temperature increases. The creep-recovery behavior of high tenacity and super low-shrinkage industrial polyester yarns at various stress levels was investigated by Chen et al. [9]. Prevorsek et al. [10] have shown how the morphological structure and properties of poly(ethylene terephthalate) fibers can be affected by a thermal contraction. Chen et al. also investigated the effect of different polycondensation methods of HT polyester fiber on its structure and properties [11].

According to the study conducted by Cho et al., heat treatment of PET fibers under free relaxation conditions formed micro-crystals in the amorphous region, which is responsible for the change in the mechanical property of the polyester fibers [12]. Liu et al. [13] have shown, in their paper, the influence of the thermal annealing process on the thickness of amorphous and crystalline region of poly(ethylene terephthalate).

The textile materials produced from high tenacity industrial polyester fibers have a wide application in mechanical rubber goods to reinforce conveyor belts, v-belts, hoses, and tires due to their high strength, high modulus of elasticity, resistance to stretching, and high thermal stability [14,15,16]. Among industrial polyester yarn types, high tenacity polyester yarn is used to produce a woven fabric for a conveyor belt reinforcement application. During the conveyor belt production, the rubber, reinforced with a textile fabric, is subjected to a vulcanization process at a high temperature for a specific aging time. Barburski has analyzed the mechanical properties of a conveyor belt reinforced by a woven fabric produced from a high tenacity polyester and polyamide yarns at three different production stages of the conveyor belt [17]. In another paper, he also investigated the effect of heat treatment on the internal structure of fabrics during the conveyor belt’s vulcanization process [18,19].

Even though numerous studies have been conducted on the properties of industrial polyester fiber and the carcass of conveyor belts, the effect of thermal aging on the industrial polyester yarn’s mechanical property has not received much attention from researchers. The main aim of this study is to investigate the effect of thermal aging parameters, which are temperature and aging time, on the properties of high tenacity polyester yarn. The aging temperature and duration of aging used in this work are based on the aging temperature and time used to vulcanize textile reinforced conveyor belts.

## 2. Materials and Methods

### 2.1. Materials

A pre-activated high tenacity polyester (poly(ethylene terephthalate)) yarn, used to produce conveyor belt fabric carcasses, was obtained from Kordárna Plus a.s. Company, Czech Republic. The properties of the basic yarn (110 tex) supplied by Kordárna Plus a.s. Company are provided in Table 1.

The PET yarn samples with a linear density of 220 tex, 440 tex, 660 tex, and 990 tex were obtained by twisting the basic yarn (110 tex) with 90, 60, 60, and 60 turns per meter (TPM) in the S-direction, respectively.

### 2.2. Yarn Aging

**Figure 1:**
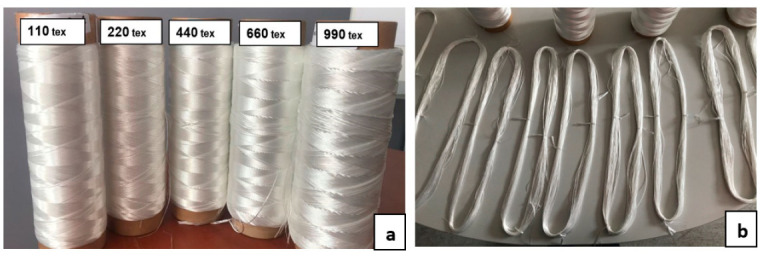
(**a**) Yarn samples in bobbin form; (**b**) Yarn samples in hank form.

The industrial polyester yarn samples were prepared in the form of hank to facilitate the fibers’ thermal aging, as shown in Figure 1b. The yarns prepared in the hank form were subjected to thermal aging by putting the samples inside an industrial oven.

The oven was first overheated above the required aging temperature to avoid the temperature drop while placing the samples in the oven. The yarns were thermally aged at 140 °C, 160 °C, 200 °C, and 220 °C within the aging time of six, twelve, and thirty-five minutes. The yarns’ tensile strength and surface structural properties were tested before subjecting the samples to thermal aging and after thermal aging. The effect of thermal aging temperature and aging time on the tensile strength, elongation, and surface structure of high tenacity PET fiber was investigated in this study.

### 2.3. Tensile Strength Testing

**Figure 2:**
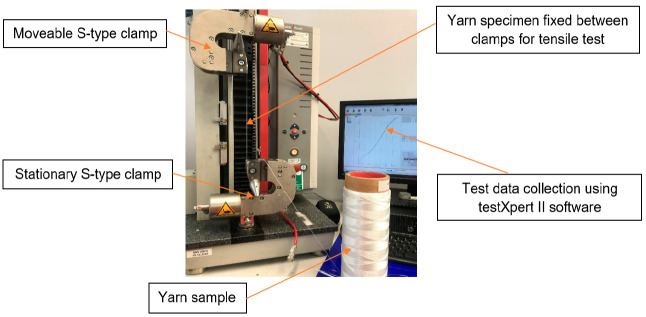
Zwick/Roell tensile tester.

The tensile properties of the polyester yarns were measured on a Zwick/Roell tensile tester of a 2.5 kN load (Figure 2), with a gauge length of 250 mm, and a constant rate of extension of crosshead speed of 250 mm/min under standard laboratory conditions. An S-type clamp was used during the test. From each type of yarn sample, twenty yarn specimens were tested for each stage of thermal aging. The tensile test was conducted, and the mean values were reported in accordance with ISO 2062:2009 [20].

### 2.4. Scanning Electron Microscope Observation

The high tenacity polyester fiber’s surface structure before and after thermal aging was examined using a scanning electron microscope (SEM). The test was carried out on 110 tex and 660 tex of yarn samples, and the surface structure of the fibers was presented.

## 3. Results and Discussion

### 3.1. Unaged High Tenacity Polyester Yarn Tensile Strength Test Results

**Figure 3:**
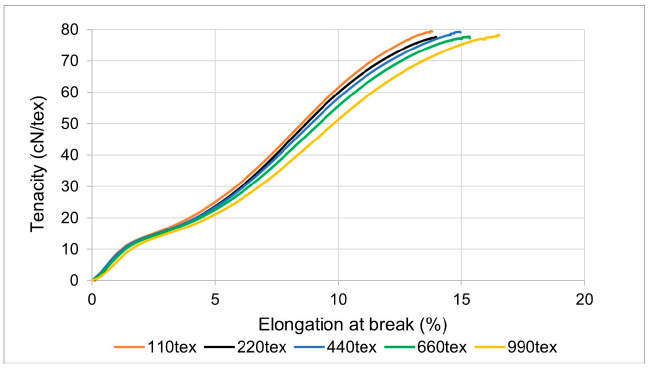
Stress–strain diagram of unaged high tenacity polyester yarn.

The tensile test results of the polyester samples before subjecting the yarns to thermal treatment are shown in Figure 3.

The tensile strength test result of the unaged high tenacity (HT) polyester yarn shows that the increase in the yarn’s linear density had no significant change (±1 cN/tex) on the yarn’s tenacity, as shown in Figure 3. The stress–strain curve also signifies that all tested HT polyester yarn samples had similar behavior in the curve’s elastic range. However, in the curve’s plastic range, the yarn sample with a lower linear density exhibited a slightly lower percentage elongation. The elongation increased with the increase in the yarn’s linear density. Nevertheless, the percentage elongation difference in the yarn samples is insignificant (±2%).

### 3.2. Comparison of 110 Tex High Tenacity Polyester Yarn Tensile Properties in a Different Aging Time and Temperature

**Figure 4:**
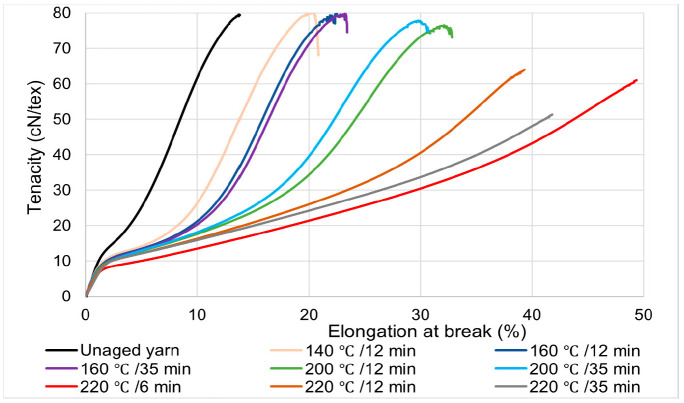
Comparison between stress–strain diagram of unaged and thermally aged 110 tex of high tenacity polyester yarn.

The tensile strength test results of the 110 tex PET yarn samples aged under different aging time and temperatures are shown in Figure 4. The results indicate that the tensile properties of high tenacity polyester yarn were changed after thermal aging compared to the unaged yarn sample. The property change was observed with both the variation of aging temperature and time.

In the tenacity vs. percentage elongation curve shown in Figure 4, it was observed that all samples had the same characteristics in the elastic range of the curve regardless of the thermal aging conditions. However, in the plastic range, the response of the sample to an applied load varied with the thermal aging conditions. The changes that appeared in the yarn sample properties due to thermal aging conditions are discussed below in relation to the unaged yarn curve shown in black. There were no significant tenacity differences observed on the yarn samples aged at 140 °C and 160 °C, regardless of the aging time. Furthermore, a minor change (±0.55 cN/tex) in tenacity was observed on the yarn sample aged at 200 °C, compared to the unaged yarn tenacity.

The overall result indicates that the yarn aged at a higher temperature with a lower aging time (temperature 220 °C for the 6-min of aging time) displayed an almost five times higher percentage elongation at break compared to the unaged yarn sample. Elongation of the textile material is a crucial parameter in the reinforcement of the conveyor belt, as it has a direct impact on the belt’s dimensional stability. Thus, the elongation of the yarn needs to be comprehensively examined. The unaged yarn had a relatively high tenacity compared to the samples subjected to thermal aging above 200 °C. This phenomenon signifies that exposure of the thermoplastic yarns (PET) to a higher temperature deteriorates the structure of the fibers; this leads to the yarn degrading easily under a minor applied load [21]. In Figure 4, only the stress–strain of the 110-tex polyester yarn sample is shown; for the remaining samples, the results are presented in the following sections of the paper in the form of bar charts.

### 3.3. Effect of Temperature on the Polyester Yarn’s Tenacity

**Figure 5:**
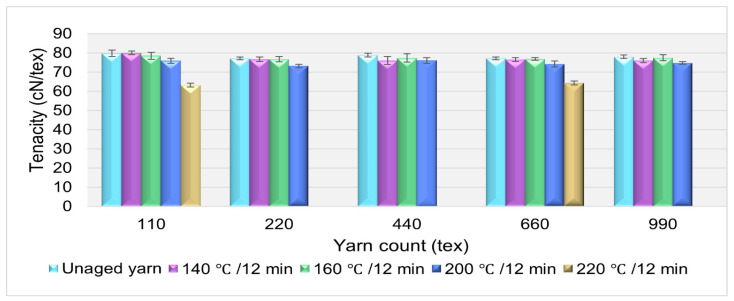
Effect of aging temperature on the tenacity of HT polyester yarn.

The effect of temperature on the polyester yarn’s tenacity was investigated by aging the yarn under different aging temperatures within a constant aging time. The thermal aging of yarn at 220 °C was performed for 110 tex and 660 tex sample types. As shown in Figure 5, the result reveals that when the yarns were subjected to aging temperatures from 140 to 200 °C for 12-min of aging time, an insignificant change was observed (±0.55 cN/tex) in the yarn’s tenacity, despite the change in the yarn’s linear density. Nevertheless, as the aging temperature rose to 220 °C, a tenacity loss of 20.84% was noted in comparison to the unaged yarn.

**Figure 6:**
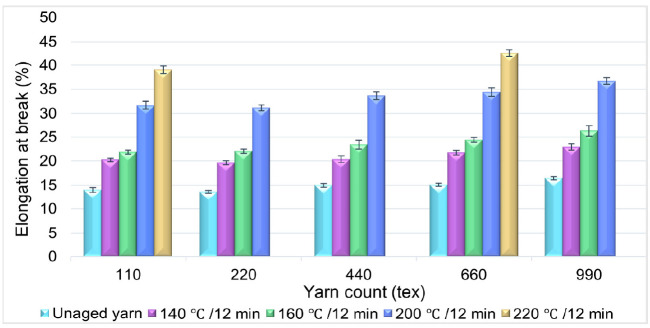
Effect of aging temperature on the percentage elongation of HT polyester yarn.

This signifies that an increase in aging temperature can soften the yarn’s polymeric structure and reduce the ability of the yarn to withstand external forces. However, this increases the percent elongation of yarn under a meager external load, as shown in Figure 4 and Figure 6.

### 3.4. Effect of Temperature on Percentage Elongation of HT Polyester Yarn

Contrasting to the tenacity of the yarn, a significant change in the percentage of elongation at break was noted with the increase in aging temperature while aging duration was constant, as shown in Figure 6. Many researchers [4,5,9,13,14] have presented that thermal aging of polyester yarn, above its glass transition temperature and below the melting point, results in the modification of the fiber’s internal structure; this drastically changes the mechanical property of the yarn. As the temperature approaches the melting point of the polyester fiber (260 °C) [22], the ability of the polymer molecules rearrangement is high, and this phenomenon facilitates the elongation of yarn under a meager applied load. The polyester fiber’s internal structure modification under thermal treatment has been investigated in past studies [10,13,23,24], but these analyses have not included how the rearrangement of the internal structure affects the percentage elongation of polyester yarn. The thermal aging of yarn at 220 °C was performed only for the 110 tex and 660 tex sample types.

The percentage elongation increased with the rise in aging temperature. The results of the yarns aged at 140 °C and 160 °C show a minor percentage elongation change, which was, on average, 2.64%, but as the temperature rose above 160 °C, the percentage elongation change was elevated. From the bar chart shown in Figure 6, it can be concluded that the elongation of the high tenacity polyester yarn increased as the aging temperature rose to 220 °C. Earlier studies, conducted by various researchers, also show that the elongation of polyester fibers and yarns increases with the increase in temperature [23,25]. However, the temperature range at which the percent of elongation increases or decreases depends on the type of polyester fiber.

### 3.5. Effect of Aging Time on the Polyester Yarn’s Tenacity

**Figure 7:**
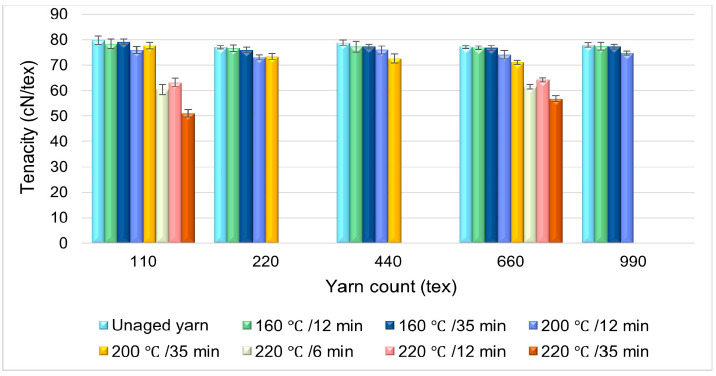
Effect of aging time on the tenacity of HT polyester yarn.

The effect of thermal aging time on the polyester yarn’s tenacity was also investigated by varying the aging time while the aging temperature was constant. Figure 7 shows the influence of aging time, under the aging temperatures of 160, 200, and 220 °C, on the yarn’s tenacity. The results reveal that the tenacity of the yarn aged for 12 and 35 min under 160 °C of the aging temperature was almost the same, with only an average tenacity decrement of ±0.02 cN/tex as the aging time increased from 12 to 35 min. The result of the yarn samples aged under 200 °C for 12 and 35 min showed a 1.11 cN/tex decrement of tenacity as the aging time varied from 12 to 35 min regardless of the yarn’s linear density.

To further investigate the effect of aging time, the yarn samples of 110 tex and 660 tex were aged under 220 °C for six, twelve, and thirty-five minutes of aging time. The tenacity of the yarns aged for 12 min at 220 °C was slightly higher in comparison to the samples aged for six and thirty-five minutes. A lower tenacity was obtained for the yarn aged at a higher temperature (220 °C) for a longer aging time (35 min). The experimental analysis shown in Figure 7 indicates that the effect of aging duration on the tenacity of yarn is influenced by the aging temperature range. For yarns aged below 200 °C, there were no considerable changes observed even though the aging duration was incremented from 12 to 35 min. This indicates that polymer chains’ weakening in the fiber structure is negligible when the aging temperature is far below the polyester fiber’s melting point. The study conducted by Liu et al. in 2016 on high-modulus low-shrinkage poly(ethylene terephthalate) fiber also shows that there is no evident change observed in the structure of the polyester fiber below 200 °C of aging temperature [13].

### 3.6. Effect of Aging Time on the Elongation of HT Polyester Yarn

**Figure 8:**
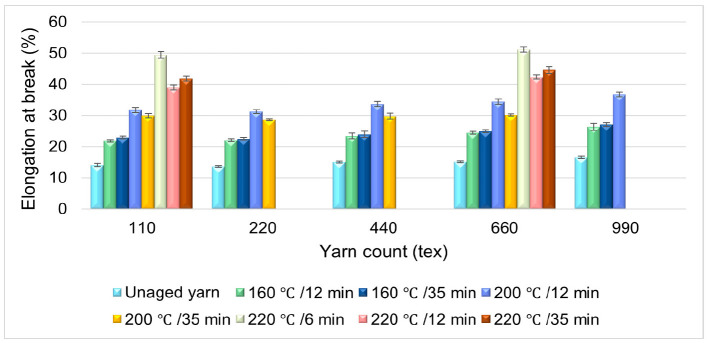
Effect of aging time on the percentage elongation of HT Polyester yarn.

The effect of aging time on the percentage elongation of polyester yarn is shown in Figure 8. The bar graph shown in Figure 8 illustrates the comparison of yarn samples aged at different aging times. The first light-blue colored bar graph shows the percentage elongation of the unaged yarn. The light-green and navy colored bar graphs are the results of the yarn samples aged at 160 °C for different aging times; the percentage elongation difference observed at this specific aging temperature, because of aging time variation, is insignificant for all samples. The medium-slate blue and orange colored bar graphs show the percentage elongation of the yarn samples aged at 200 °C for twelve and thirty-five minutes of aging time; the results reveal that as the aging time increased at this temperature (200 °C), the percentage elongation of the yarn decreased, on average, by 3.93%. The 110 tex and 660 tex samples were aged at 220 °C for six, twelve, and thirty-five minutes of aging time, and the highest percentage elongation was observed on yarn samples aged at 6 min, which is almost five times higher in comparison to the unaged yarn.

### 3.7. Effect of Aging Time and Temperature on the Percentage of Elongation and Tenacity

**Figure 9:**
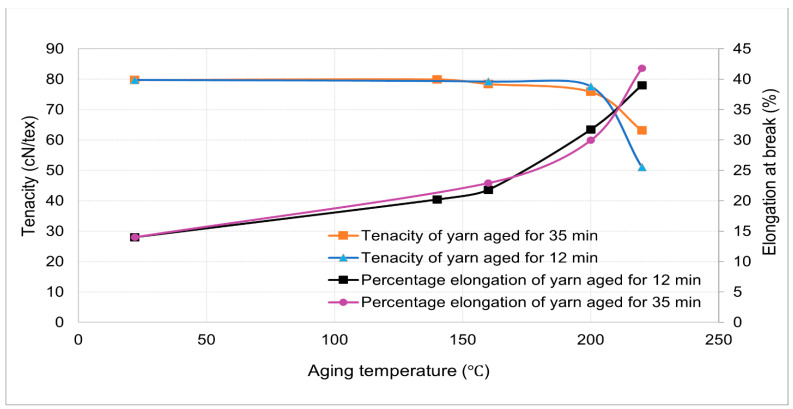
Effect of aging time and temperature on the percentage elongation and tenacity of 110tex HT Polyester yarn.

The overall correlation of aging time, temperature, tenacity, and elongation of yarn is shown in Figure 9. The analysis demonstrates that the tenacity and percentage elongation of the yarn were inversely correlated. Additionally, the aging time affected both the tenacity and percentage elongation of the yarn, but the effect depends on the aging temperature.

### 3.8. Structural View of Unaged and Aged HT Polyester Yarn

**Figure 10:**
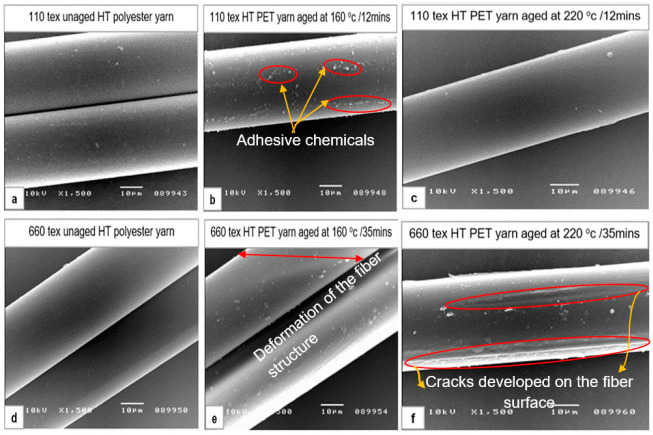
Surface structural view of high tenacity polyester fiber using scanning electron microscopy (SEM). (**a**) 110 tex unaged HT polyester yarn; (**b**) 110 tex HT polyester yarn aged at 160 °C for 12 min; (**c**) 110 tex HT polyester yarn aged at 220 °C for 12 min; (**d**) 660 tex unaged HT polyester yarn; (**e**) 660 tex HT polyester yarn aged at 160 °C for 35 min; (**f**) 660 tex HT polyester yarn aged at 220 °C for 35 min.

The surface structural observation of high tenacity polyester fiber was carried out using a scanning electron microscope (SEM) on 110 tex and 660 tex yarn samples for a selected aging temperature and aging time. The images in Figure 10a–d signify that there was no considerable surface structural change observed on the fibers’ surface. Nevertheless, the adhesive chemicals on the surface of the pre-activated HT polyester yarn were observed in the form of little spots grouped together on the sample aged at 160 °C for 12 min, as shown in Figure 10b. As the aging temperature was elevated to 220 °C for 35 min (Figure 10c), the adhesive chemicals fully melted and a smooth fiber surface was observed. Additionally, minor surface deformation was detected on a yarn sample aged at the temperature of 160 °C for 35 min, as shown in Figure 10e. Moreover, on the yarn sample aged at a higher temperature (220 °C) for a longer aging time (35 min), surface damage (cracks and spots on the surface) was observed, as shown in Figure 10f, and because of this, the lowest tenacity of yarn was obtained at this aging condition, as shown in Figure 7.

## 4. Conclusions

The effect of thermal aging parameters on the tenacity, elongation, and surface structural properties of the pre-activated high tenacity polyester yarn was investigated by conducting thermal aging on different polyester yarn samples under various aging temperatures and aging time. The following conclusions can be drawn from the study.

- The stress–strain curve of all polyester samples included in this work shows that the response of the yarns to a given load in an elastic region of the curve is similar regardless of the thermal aging conditions and linear density of the yarn. However, the plastic region characteristics of the curve are reliant on the linear density of the yarn, aging temperature, and aging time.
- The polyester yarn samples subjected to thermal aging at the temperature of 140 °C and 160 °C at 12 min and 35 min have not shown any tenacity difference compared to the unaged yarn sample. Nevertheless, the tenacity of the polyester yarn was lowered as the temperature rose above 200 °C. Compared to the unaged yarn sample, the tenacity of the yarns aged at 200 °C and 220 °C for 12 min dropped by 4.28% and 18.45%, respectively. Moreover, the yarn aged at 220 °C for 35 min displayed, on average, a 30.97% lower tenacity compared to the unaged yarn.
- The elongation of the polyester yarn samples increased with the increase in temperature, aging time, and the yarn’s linear density, and an almost five times higher elongation was obtained for the yarn aged at 220 °C for 6 min compared to the unaged yarn.
- The fiber’s surface structure was also affected as the yarn underwent thermal aging at a higher temperature (220 °C) for a longer aging time (35 min).

Overall, the thermal aging parameters significantly influence the yarn’s mechanical and surface structural properties when the yarn is subjected to an aging temperature above 220 °C. Additionally, the tenacity and elongation of the yarns are inversely proportional as the aging temperature rises. Further experiments are also in progress related to the effect of thermal aging on the internal structure of high tenacity polyester fibers and on fabrics made from HT polyester yarns for conveyor belt reinforcement purposes.

## Figures and Tables

**Table 1:** Properties of 110 tex pre-activated high tenacity (HT) polyester yarn.

Yarn Type	Property				
	Linear Density (tex)	Breaking Force (N)	Breaking Tenacity (cN/tex)	Elongation at Break (%)	Hot Air Shrinkage (%)
High Tenacity Polyester	110	89.9	81.0	13.5	5.50

## Data Availability

The data presented in this study are available on the request from the corresponding author.
